# Design and Implementation of a Low-Noise Analog Front-End Circuit for MEMS Capacitive Accelerometers

**DOI:** 10.3390/mi17030378

**Published:** 2026-03-20

**Authors:** Keru Gong, Jiacheng Li, Xiaoyi Wang, Huiliang Cao, Huikai Xie

**Affiliations:** 1 School of Integrated Circuits and Electronics, Beijing Institute of Technology, Beijing 100081, China; 3120231352@bit.edu.cn (K.G.); 3120225693@bit.edu.cn (J.L.); xiaoyiwang@bit.edu.cn (X.W.); caohuiliang@bit.edu.cn (H.C.); 2 Chongqing Institute of Microelectronics and Microsystems, Beijing Institute of Technology, Chongqing 400000, China; 3 Engineering Research Center of Integrated Acousto-Opto-Electronic Microsystems, Ministry of Education of China, Beijing 100081, China; 4 East China Institute of Photo-Electron lC, Bengbu 233030, China

**Keywords:** analog front-end electronics, MEMS, accelerometer, optical image stabilization, CMOS

## Abstract

This paper presents a low-noise analog front-end (AFE) integrated circuit (IC) circuit for capacitive micro-electromechanical system (MEMS) accelerometers that can be used for optical image stabilization (OIS) in various optical imaging systems. The AFE circuit design features a fully differential chopper stabilization technique that efficiently minimizes low-frequency 1/f noise and parasitic coupling. The AFE circuit chip is fabricated in a 0.18 μm complementary metal-oxide-semiconductor (CMOS) technology and co-packaged with an x-axis capacitive MEMS accelerometer based on a silicon-on-glass (SOG) process. The SOG accelerometer has a footprint of 1000 μm × 950 μm. The packaged system demonstrates a sensitivity of 342 mV/g and a nonlinearity of 1.1% between −1 g and +1 g, a dynamic range of 88 dB, and an equivalent noise floor of 14 $\mu g/\sqrt{Hz}$.

## 1. Introduction

Image stabilization (IS) technology is essential for portable electronic imaging devices. For instance, camera movement during exposure in photography may lead to blurred images, due to inherent body motions or vibrations in the imaging settings [1]. IS technology can be categorized into three primary types: digital image stabilization (DIS), electronic image stabilization (EIS), and optical image stabilization (OIS) [2]. Both DIS and EIS require substantial memory and computing resources. In contrast, OIS utilizes micro-electromechanical system (MEMS) accelerometers and gyroscopes to detect motion, facilitating direct adjustments of the lenses or even the imaging sensors in the camera, thus minimizing memory and computing resource usage [3,4]. Moreover, OIS can mitigate image blur in low-light conditions, addressing the blurring challenges posed by EIS and DIS in dim conditions [5]. Furthermore, OIS prevents image cropping at the edges, maintaining the integrity and beauty of the original scene while providing enhanced stability [6].

MEMS accelerometers function as the primary sensor in an OIS system, detecting the camera’s linear motion, tilt angle, and spatial vibration. Their performance directly affects the effectiveness of IS technology. OIS imposes stringent requirements on MEMS accelerometers, including high resolution, low noise, compact size, and low power consumption [7]. Capacitive MEMS accelerometers provide high sensitivity, low noise and low power consumption [8,9]. The analog front-end (AFE) readout circuit is a key component that determines the performance of capacitive MEMS accelerometers [10]. Therefore, designing a low-noise, low-power AFE readout circuit is essential for achieving high-performance accelerometers.

The AFE readout circuit for capacitive accelerometers is typically implemented using either continuous-time [10,11,12,13] or discrete-time [14,15,16] architectures. Continuous-time circuits commonly utilize chopper modulation techniques to effectively reduce noise in the baseband. In contrast, discrete-time readout circuits primarily use switched-capacitor technology, often in conjunction with Σ-Δ modulators [17], which is limited by the effects of switching noise folding. However, conventional continuous-time architectures contend with critical design trade-offs. Firstly, establishing the necessary high-impedance direct current (DC) biasing for sensing often requires large passive components, which will result in a significant increase in chip area [10,18]. Secondly, continuous-time architectures typically face a direct conflict between noise and power, and suppressing the thermal noise floor generally necessitates increasing the bias current, thereby elevating power consumption [13,19].

In this study, an optimized continuous-time readout circuit topology is adopted to address the OIS system’s requirements for high precision, low noise, and reduced power consumption. Specifically, metal-oxide-semiconductor (MOS)-bipolar device pseudo-resistors are implemented to stabilize the input DC bias voltage efficiently. Furthermore, a two-stage dual chopper structure is employed to effectively balance low-noise performance and power consumption. This synergized approach optimizes the overall trade-off between chip area, noise, and power efficiency. Although the circuit techniques used in this work are based on established design methods, the contribution of this study lies in their application-oriented integration and optimization for the target sensor readout interface. Rather than introducing a single new circuit block, this work focuses on a practical form of AFE implementation that simultaneously addresses weak-signal amplification, low input-referred noise, low offset, and stable operation under the constraints of OIS applications. The main contributions are as follows. First, a dual-chopper AFE architecture is adopted and optimized according to the characteristics of the target sensor. Second, the front-end and back-end stages are co-designed to achieve a practical trade-off among gain, noise, bandwidth, power consumption, and circuit complexity. Third, the proposed complementary metal-oxide-semiconductor (CMOS) AFE is experimentally validated together with the MEMS accelerometer, and the measured results demonstrate competitive performance for OIS applications.

This work develops an AFE readout circuit for a capacitive MEMS accelerometer that is based on a silicon-on-glass (SOG) process, specifically designed for OIS applications. Section 2 introduces OIS systems and the circuit design specifications. Section 3 presents the mechanical structure of the accelerometer, subsequently addressing the comprehensive circuit architecture design and particular modulate designs, including operational amplifiers. Section 4 presents the experimental results, including individual circuit validation and static and dynamic acceleration testing of the capacitive MEMS accelerometer.

## 2. System Overview and Design Objectives

### 2.1. OIS System

**Figure 1:**
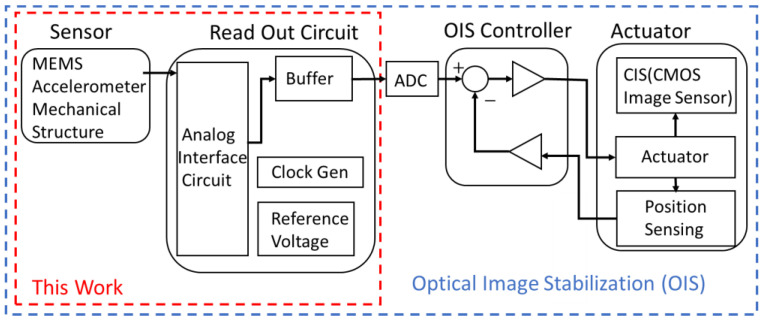
Overall block diagram of the OIS system.

A typical OIS system with closed-loop control is illustrated in Figure 1. The stabilization process begins with detecting the movement of the camera in the imaging system. Integrated micro-sensors, specifically an MEMS accelerometer and a gyroscope, continuously monitor vibrations and angular displacements of the camera [20].

The conversion of the physical motion of the camera into executable electrical signals is realized by the forementioned AFE readout circuit, which constitutes the primary contribution of this study. Simultaneously, for accurate actuation control, a specialized position-sensing circuit embedded in the micro actuator provides continuous feedback on the image sensor’s real-time displacement. The controller continuously compares the motion detected by the sensors with the actuator’s actual position, calculating and applying the precise drive signal to physically displace the optical component and thereby eliminate image blur.

### 2.2. Design Objectives

The implementation of OIS imposes specific performance requirements on the integrated accelerometer to ensure precise vibration detection. Current studies identify hand tremor as an oscillating signal with a typical amplitude (θ) of less than 0.5 degrees [5]. The acceleration is proportional to the square of the frequency. To determine the minimum detectable signal threshold, the worst-case scenario at the lowest typical vibration frequency ($f_{min}\;=\;1\;Hz$) is considered [21]. The minimum linear acceleration, $a_{min}$ can be calculated as:

(1) $$a_{min}\;=\;r\;\times \;\theta \;\times \;{\left(2\pi f\right)}^{2}$$

where r is the rotational radius and is 0.05 m, θ  is the vibration angle, and f is the vibration frequency. Considering the physical parameters of typical handheld imaging devices such as smartphones, the typical values for r, θ  and f are 0.05 m, 0.5° and 1 Hz, respectively [5]. Based on the minimum linear acceleration, with a signal-to-noise ratio (k) requirement of 10 and a typical hand shake frequency bandwidth (BW) of 30 Hz [21], the upper limit of the noise density is calculated using the following formula to be 32 $\mu g/\sqrt{Hz}$:

(2) $$a_{noise}\leq \;a_{min}/\left(k\times \sqrt{BW}\right)$$

Regarding the frequency response, although the dominant energy of hand motion is concentrated below 30 Hz, the bandwidth of the sensor should be 5 to 10 times the signal bandwidth. To minimize phase lag within the signal band and ensure loop stability, the sensor bandwidth is specified at 200 Hz, effectively covering the signal spectrum while filtering out high-frequency mechanical resonance [21,22]. Accordingly, a target bandwidth of 200 Hz has been selected for this design. Regarding the acceleration range, the accelerometer must withstand accelerations ranging from −1 g to 1 g [5]. This covers most vibration amplitudes that cause image blur in typical handheld scenarios while also compensating for the 1 g acceleration during free fall when the camera is accidentally dropped. Finally, to meet the integration constraints of modern portable devices, the chip area of the entire AFE system should be less than 1 mm^2^ to enable compact integration within space-constrained camera modules. These specifications are summarized in Table 1.

## 3. The AFE Circuit Design for the MEMS Accelerometer

### 3.1. The MEMS Accelerometer

**Figure 2:**
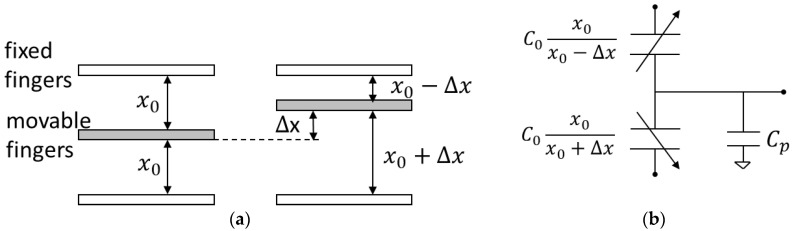
(**a**) Schematic diagram of comb teeth structure; (**b**) equivalent capacitance diagram.

A capacitive MEMS accelerometer can be fundamentally characterized as a spring-mass-damper system [9]. The schematic diagram of the comb tooth structure and the equivalent capacitance diagram is presented in Figure 2. When the frequency is markedly below the resonance frequency, the sensitivity of a mechanical device can be calculated as the ratio of displacement to acceleration.

(3) $$S_{M}=∆x/a=m/k=1/\omega_{0}^{2}$$

Mechanical damping originates from two sources: structural damping and viscous damping due to gas fluxes. The structures consist of silicon and glass, both of which are high-Q materials exhibiting minimal structural damping. Consequently, the squeeze-film damping between the fingers of a parallel-plate capacitor is the primary damping mechanism.

The pressure film damping in the detection direction can be written as [23]:

(4) $$b=N\frac{\mu Lh^{3}}{x^{3}}\beta \left(\frac{W}{L}\right)$$

where N is the total number of comb teeth, L is the length of the comb teeth, and h is the device thickness. Air kinematic viscosity (μ) at standard atmospheric pressure is $1.832\times 10^{-5}\;Pa\cdot s$. The $\beta \left(\frac{W}{L}\right)$ is the correction factor.

Air damping directly results in Brownian noise, produced by the Brownian motion of surrounding molecules. The Brownian noise floor is expressed as follows:

(5) $$a_{nm}\left(f\right)=\frac{\sqrt{4kTb}}{9.8m}\;\;\;\;\;(g/\sqrt{Hz})$$

**Figure 3:**
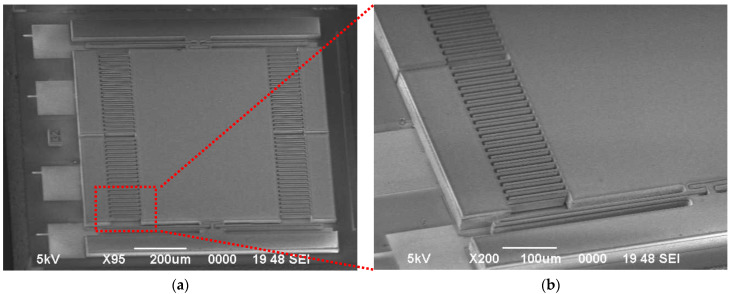
(**a**) SEM photograph of the accelerometer; (**b**) SEM photograph of comb fingers [24].

This device is constructed with a SOG bonding technique [24]. Figure 3 shows an SEM photograph of the accelerometer and a magnified view of the comb fingers. The high-resistivity (>$10^{10}\;\Omega \cdot cm$) glass substrate functions largely as mechanical support and an electrical isolation medium within this structure. Due to the non-conductive nature of glass, a parasitic conductive pathway does not exist between the moveable silicon structure and the external environment in the absence of a specific metal ground layer beneath the substrate. As a result, the principal origin of parasitic capacitance in the device is markedly diminished. Only limited coupling from the edge electric field of the moveable structure persists, with values so low (approximately sub-nanofarads) that they can be considered inconsequential. The detailed parameters of this SOG accelerometer are summarized in Table 2.

### 3.2. AFE Circuit Design for the SOG Accelerometer

The design of the AFE circuit is based on the MEMS accelerometer’s parameters listed in Table 2, as well as the performance criteria for the accelerometer sensor specified by the OIS system in Table 1. The main design goals are to ensure that the system’s overall bandwidth, acceleration range, and noise performance satisfy application requirements.

#### 3.2.1. Overall Architecture

**Figure 4:**
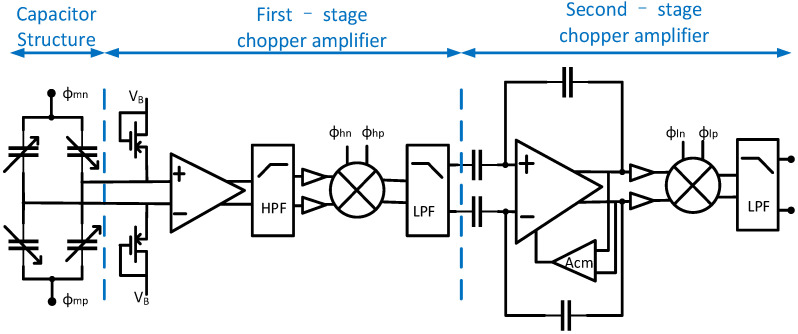
Overall circuit architecture.

The overall circuit architecture is shown in Figure 4. Based on the mapping and design derivation from sensor structure parameters to circuit specifications, the overall architecture design primarily considers the following four aspects.

**Figure 5:**
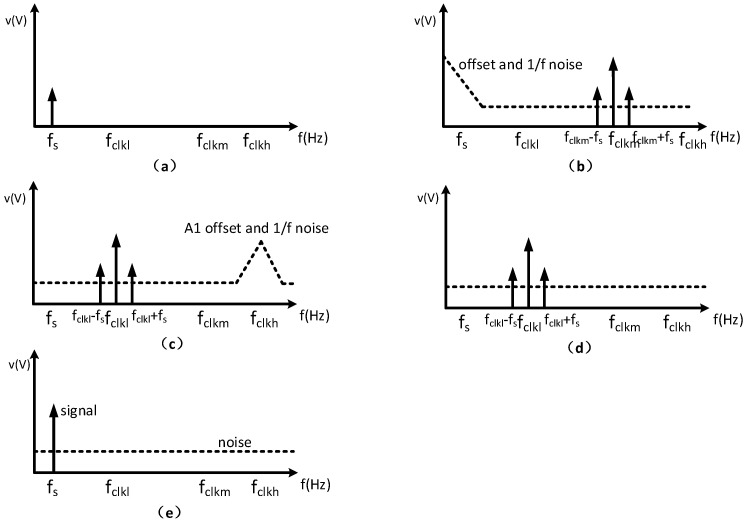
(**a**) Sensor output at baseband; (**b**) signal upconverted by the mixed clock clk_m_; (**c**) signal translated to the clk_l_, the offset and 1/f noise are shift to clk_h_; (**d**) signal after A_2_ and noise filtering; (**e**) final baseband output after demodulation by clk_l_.

(a) Continuous-time architecture: this study employs a continuous-time circuit architecture, which prevents noise folding and provides essential advantages over discrete-time designs. In discrete-time circuits, broadband noise and interference sources are integrated into the signal bandwidth during periodic sampling, resulting in heightened in-band noise [10]. The continuous-time architecture eliminates the noise folding issue by removing the sampling process.
(b) High-impedance DC biasing scheme: to establish a stable DC operating point without area-consuming passive resistors, an MOS-bipolar pseudo-resistor scheme is implemented. Utilizing sub-threshold leakage, this structure provides ultra-high resistance, significantly reducing the chip area compared to conventional solutions.
(c) Chopper stabilization technology: this circuit employs chopper stabilization technology to significantly suppress low-frequency noise and offset. By modulating the input signal to a chopper frequency to amplification, then demodulating it back to baseband, inherent 1/f noise and DC offset are upconverted and filtered out [25]. Figure 5 illustrates the spectral shift process of chopper modulation. Through chopper stabilization technology, the circuit meets the ultra-low noise requirements for OIS applications.
(d) Dual-chopper amplifier: The dual-chopper architecture shown in Figure 4 is used to balance impedance matching, noise reduction, and power consumption. Since the capacitive MEMS accelerometer has frequency-dependent impedance characteristics, a relatively high modulation frequency of 500 kHz is adopted in the first chopper stage to improve the interface between the sensor and the front-end amplifier. This high chopping frequency also shifts the signal away from the low-frequency region and reduces the influence of 1/f noise. However, operating the entire amplifier chain at such a high frequency would require a larger bandwidth and lead to higher power consumption. Therefore, a second chopper stage operating at 20 kHz is introduced so that, after the initial high-frequency modulation and amplification, the signal can be translated to a lower frequency for the following low-bandwidth and low-power amplification stages. In this way, the proposed dual-chopper topology provides a practical trade-off among sensor interfacing, noise performance, and power efficiency.

The proposed circuit utilizes three pairs of chopper clocks: a high-frequency clock clk_h_ at 500 kHz for the first-stage chopping amplifier, a low-frequency clock clk_l_ at 20 kHz for the second-stage chopping amplifier, and a mixed clock clk_m_, generated by the xor of clk_h_ and clk_l_, for the initial sensor modulation. The low-frequency chopping clock of 20 kHz is selected to be much higher than the flicker-noise corner frequency of the input transistors, thereby ensuring effective suppression of 1/f noise.

Figure 5 illustrates the frequency translation process of the proposed dual-chopper readout circuit. The differential output of the capacitive sensor bridge is first modulated by the mixed clock clk_m_, generated by the xor of clk_h_ and clk_l_, so that the low-frequency sensor signal is shifted from baseband to a higher-frequency region. The modulated signal is then amplified by the first-stage amplifier A_1_, while the offset and 1/f noise of A_1_ remain at low frequency. After that, the output of A_1_ is multiplied by clk_h_, which translates the useful signal to the frequency band associated with clk_l_ and simultaneously shifts the offset and 1/f noise of A_1_ to the vicinity of clk_h_. The signal is subsequently amplified by the second-stage amplifier A_2_, and the noise components around clk_h_ are suppressed by filtering. Finally, the output of A_2_ is multiplied by clk_l_ to demodulate the signal back to baseband, whereas the residual offset and low-frequency noise of the back-end stage are shifted out of band and removed by the final low-pass filter.

#### 3.2.2. Module Design

**Figure 6:**
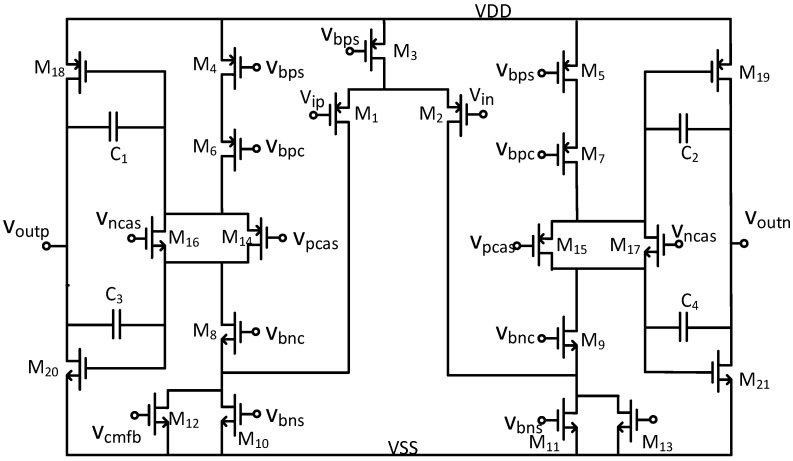
Operational amplifier block diagram.

Given the system’s comprehensive circuit design objectives, the fundamental operational amplifier must employ a fully differential architecture while meeting essential performance parameters, including high DC gain, wide unity-gain bandwidth, elevated slew rate, and minimal input noise [26,27]. Accordingly, this paper presents the design of a two-stage operational amplifier that integrates a folded-cascode first stage with a class AB output stage, as shown in Figure 6.

The two-stage architecture was adopted to satisfy the high-gain, low-noise, and large-output-swing requirements under a 1.8 V supply. A single-stage folded-cascode amplifier can provide relatively high gain, but its output swing is limited by the stacked transistors, making it difficult to meet both the gain and swing requirements within the power budget of this work. Therefore, the first stage is optimized for high gain and low noise, while the second stage provides large output swing and improved slew rate.

To achieve a DC open-loop gain exceeding 100 dB, the first stage employs a folded-cascode structure as the primary gain element. The PMOS input pair (M1–M2) is sized with W/L = 64 μm/1 μm and biased at 10 μA. This configuration minimizes the input-referred noise. The cascode transistors (M4–M5: W/L = 16 μm/1 μm, M6–M7: W/L = 16 μm/0.18 μm, M8–M9: W/L = 8 μm/0.18 μm, M10–M11: W/L = 16 μm/2 μm, M12–M13: W/L = 16 μm/2 μm) enhance the output impedance through transistor stacking, thereby enabling high voltage gain within a single stage. Nonetheless, the output swing of conventional folded-cascode structures is constrained by the number of stacked transistors. To mitigate this limitation, a class AB output stage (M14–M15: W/L = 8 μm/0.18 μm, M16–M17: W/L = 4 μm/0.18 μm, M18–M19: W/L = 80 μm/0.5 μm, M20–M21: W/L = 80 μm/1 μm) is implemented with a quiescent current controlled by 100 μA. The class AB output stage not only prevents swing loss associated with multi-stage stacking but also provides robust output current drive capability with relatively low static power consumption. This approach simultaneously facilitates a large output signal swing and a high slew rate. For stability, a compensation capacitor C = 0.8 pF is incorporated. The key design parameters of the operational amplifier are shown in Figure 6.

Compare the class AB design in this work with alternative approaches from recent literature. The review by Dei et al. [28] summarizes several approaches for switched-capacitor amplifiers. Among them, the Flipped-Voltage Follower (FVF) techniques can offer high power efficiency, but the additional active devices may increase input-referred noise. Parallel-type slew-rate-enhancement (PSRE) techniques can improve large-signal behavior while preserving small-signal operation, but they usually require auxiliary control circuits and careful dead-zone design to avoid distortion. Xin et al. [29] reported a high-efficiency two-stage amplifier using inner feedforward path compensation (IFPC) to improve current efficiency. However, this type of compensation may be more sensitive to load conditions and parasitic variations. In our application, the load capacitance includes the MEMS sensor and parasitic elements from co-packaging, and these combinations will vary with manufacturing tolerances, making robust pole-zero cancelation challenging.

Therefore, this work adopts a folded-cascode first stage followed by a class AB output stage. Compared with the above alternatives, the proposed implementation provides a good trade-off among low noise, circuit simplicity, and stability robustness. Although some reported techniques may achieve better power-efficiency metrics, our design mainly targets noise performance and reliable operation under variable load conditions.

**Figure 7:**
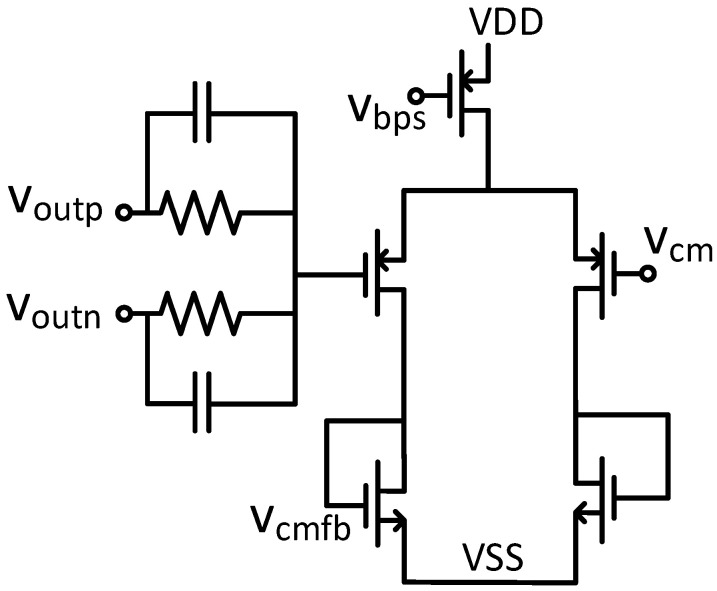
Schematic diagram of the continuous-time common-mode feedback.

While the fully differential architecture effectively suppresses common-mode interference and improves the power supply rejection ratio, the output common-mode level lacks a fixed DC offset [30]. To maintain stable common-mode operating points, the design integrates a common-mode negative feedback (CMFB) circuit. This circuit continuously monitors the output common-mode level, compares it to a reference voltage, and applies negative feedback to stabilize the output common-mode voltage at a specified value. The schematic diagram of the continuous-time common-mode feedback is shown in Figure 7.

**Figure 8:**
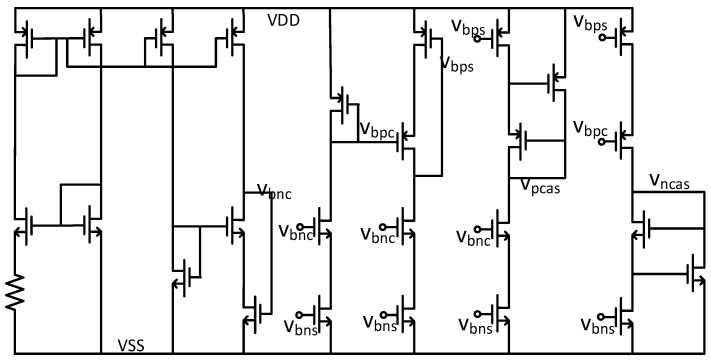
Schematic diagram of the bias circuit.

To provide a stable bias voltage for the circuit and ensure the MOSFET operates in the saturation region, the bias circuit for the entire system is illustrated in Figure 8.

#### 3.2.3. Simulation Results

In chopper amplifier circuits, MOSFETs function as chopper switches to enable signal modulation and demodulation. However, non-ideal characteristics of real switches, including on-resistance, charge injection, clock crosstalk, and thermal noise, significantly affect circuit performance. Therefore, systematic analysis of these parameters is essential for optimizing performance by selecting appropriate switch structures and dimensions.

On-resistance directly affects switching speed. To reduce resistance variations, a complementary switch configuration can be implemented. The on-resistance of complementary switches is calculated as follows:

(6) $$\begin{array}{cc} & R_{on}\;=\;R_{onP}||R_{onN}\\ & \;=\;\frac{1}{\mu_{n}C_{ox}{\left(\frac{W}{L}\right)}_{N}(V_{DD}-V_{thn})-[\mu_{n}C_{ox}{\left(\frac{W}{L}\right)}_{N}-\mu_{p}C_{ox}{\left(\frac{W}{L}\right)}_{P}]-\mu_{p}C_{ox}{\left(\frac{W}{L}\right)}_{P}|V_{thp}|} \end{array}$$

**Figure 9:**
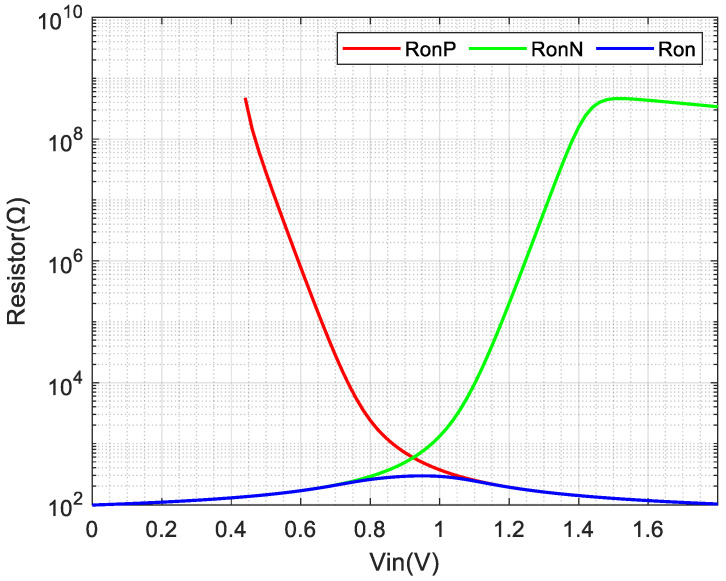
Switch on-resistance simulation image.

Figure 9 presents the impact of varying the sizes of complementary switches on the overall on-resistance, as determined by simulation analysis. The red curve represents the on-resistance when only PMOS transistors are used as switches, while the green curve corresponds to the on-resistance with only NMOS transistors. The blue curve indicates the on-resistance of the complementary switch structure in the on state. The simulation results show that the on-resistance of the complementary switch ranges from 100 Ω to 293 Ω, in line with the circuit design requirements.

**Figure 10:**
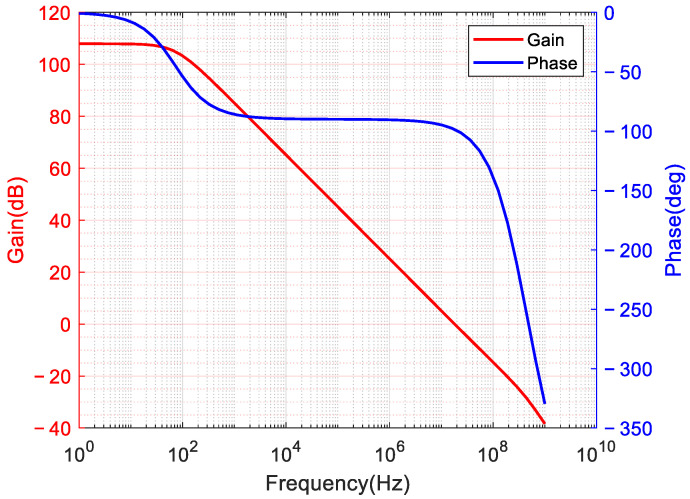
Gain-frequency and phase-frequency response curves of operational amplifiers.

The non-ideal characteristics of operational amplifiers, including DC gain, unity-gain bandwidth, slew rate, thermal noise, and 1/f noise, significantly affect circuit performance. These parameters directly determine the accuracy, speed, and noise levels in amplification circuits, necessitating careful simulation and adjustment. The performance of the constructed operational amplifier was evaluated by simulating its frequency response and transient characteristics. Figure 10 presents the operational amplifier’s response to variations in amplitude, frequency, and phase. Simulation results demonstrate that the constructed operational amplifier achieves a DC gain of 107 dB, substantially reducing amplification errors due to finite gain and making it suitable for applications requiring high accuracy. The operational amplifier exhibits a unity-gain bandwidth of 18 MHz, confirming its ability to provide sufficient gain and stability across the intended frequency range. Improving the slew rate, a key parameter for assessing the response speed to input signals, is essential to avoid prolonged settling times that can cause gain inaccuracies and signal distortion. The simulation confirmed a slew rate of 13 V/μs, indicating compliance with high-speed operational and design requirements.

The simulated power distribution of the AFE chip under 1.8 V supply is as follows. The first-stage amplifier consumes 1.045 mW, accounting for 29.3% of the total power. This stage dominates the power budget because it is designed to provide high transconductance for low-noise amplification under the 500 kHz chopping operation. The second-stage amplifier consumes 0.475 mW, corresponding to 13.3% of the total power. Owing to its lower bandwidth requirement associated with the 20 kHz chopping frequency, its power consumption is lower than that of the first stage. The chopper switches and buffers consume 0.32 mW. The bias and common-mode feedback circuits consume 0.136 mW for current reference generation and common-mode stabilization. The filters consume 0.636 mW to filter out offset and 1/f noise.

Table 3 summarizes the simulated power breakdown of the proposed AFE under a 1.8 V supply. The first-stage amplifier consumes the largest portion of the total power. The second-stage amplifier is the second major contributor, while the remaining power is distributed among the chopper switches, buffers, bias circuits, common-mode feedback circuits and filters.

The overall noise performance of the circuit is primarily limited by the operational amplifier’s 1/f flicker noise and thermal noise. To address these noise sources, specific optimization strategies were incorporated into the design. Setting the gain–bandwidth product appropriately and introducing a system-level filter structure effectively suppresses thermal noise. Additionally, the application of chopper modulation technology substantially reduces the 1/f noise component at low frequencies.

The total system noise is determined by the Brownian noise of the sensor’s mechanical structure and the noise from the circuitry. Based on the previously proposed OIS noise requirements and the Brownian noise of the sensor’s mechanical structure, the noise density of the circuitry can be estimated using the following equation.

(7) $$a_{total}^{2}=a_{brownian}^{2}+a_{circuit}^{2}$$

The overall input-referred noise of the proposed AFE is mainly determined by the first-stage amplifier. For the cascaded readout chain, the total input-referred noise can be expressed as:

(8) $$\bar{v_{noise,in}^{2}}=\bar{v_{1}^{2}}+\frac{\bar{v_{2}^{2}}}{{A_{1}}^{2}}+\frac{\bar{v_{3}^{2}}}{{A_{1}}^{2}{A_{2}}^{2}}$$

where $v_{1}$, $v_{2}$, $v_{3}$ denote the equivalent noise contributions of the first stage, second stage, and later stages, respectively, and A_1_ and A_2_ are the gains of the preceding stages. According to this relation, the first-stage noise is the dominant term in the total input-referred noise, while the contributions of the following stages are reduced when referred back to the input. Post-layout noise simulation shows that the first-stage amplifier contributes more than 80% of the total input-referred noise. Simulation results also show that the amplifier has a 1/f noise corner of approximately 350 Hz without chopping. With chopping and filtering, the low-frequency noise is effectively reduced, and the equivalent input noise density is 3 $nV/\sqrt{Hz}$.

## 4. Experiments and Results

The layout design of the AFE circuit, implemented using 0.18 μm 1P6M CMOS technology, has been finalized and successfully taped out. The test setup includes a test printed circuit board (PCB), a field-programmable gate array (FPGA) module for generating three sets of chopper clocks, a 1.8 V external desktop DC power supply, a 0.9 V reference common-mode voltage, and excitation signals with adjustable frequency and amplitude provided by the vibration table. The circuit utilizes three pairs of chopper clocks: clk_h_, a 500 kHz high-frequency control clock; clk_l_, a 20 kHz low-frequency control clock; and clk_m_, generated by the XOR of clk_h_ and clk_l_, used for initial sensor modulation. Output signals are recorded and analyzed using an oscilloscope and a signal and spectrum analyzer.

### 4.1. Noise Testing

The noise performance of the AFE readout circuit was evaluated. Chopping modulation significantly improves the voltage noise spectrum by up-converting the low-frequency 1/f noise to the chopping frequency of approximately 500 kHz. Consequently, a measured input-referred noise density of 58 $nV/\sqrt{Hz}$ is achieved within the signal bandwidth. This low noise floor is critical for detecting subtle variations in MEMS capacitance.

### 4.2. The Quasi-Static Response Testing

A static test was developed and conducted using gravitational field excitation. The gravitational acceleration of Earth (g $\approx 9.8\;m/s^{2}$) served as the reference input for known acceleration. The angle between the sensor’s sensitive axis and the neutral vector was adjusted using a high-precision turntable (JDZT240H), enabling precise control of the static acceleration component applied to the sensor’s input axis.

The sensor’s sensitive axis should be aligned parallel to the gravitational vector. The turntable angle is set to 0°, corresponding to a theoretical applied acceleration of 1 g. The turntable is then rotated in 10° increments from 0° to 180°. After the turntable stabilizes and remains steady for 10 s at each angle, the sensor’s total voltage output data is recorded. The following procedure is used to determine the acceleration input at each angle:

(9) $$a_{i}=g\cdot cos\left(\theta \right)$$

**Figure 11:**
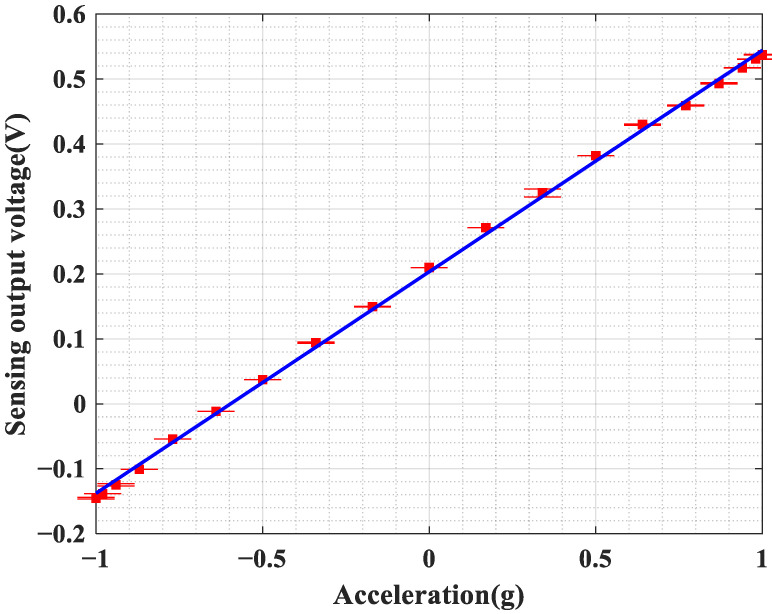
Measured quasi-static response of the accelerometer system developed in this work.

The output voltage readings for each angle should be recorded, and the average and standard deviation of the output voltage calculated. Figure 11 shows the measured quasi-static response of the accelerometer system developed in this work. The fitted curve indicates that the acceleration system exhibits an overall sensitivity of 342 mV/g. The maximum difference between the measured values at all test points and the projected values from the fitted line should be determined and expressed as a percentage of the full-scale output voltage. The system’s nonlinearity has been determined to be 1.1%.

### 4.3. The Dynamic Performance Testing

A dynamic performance test was designed and implemented to comprehensively evaluate the dynamic response characteristics of the accelerometer’s mechanical structure and AFE circuitry, including dynamic range and noise performance, under controlled vibration [31].

**Figure 12:**
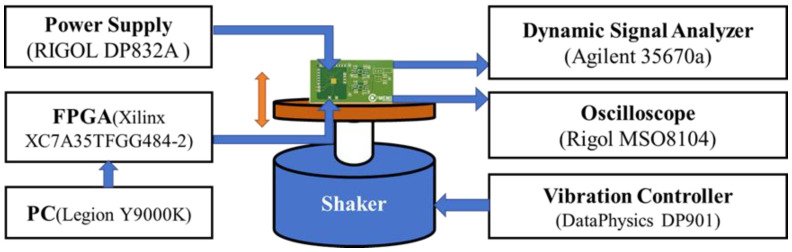
The measurement setup of dynamic test (blue arrows: signal flow; orange arrows: vibration direction of the shaker).

The connection diagram of the dynamic performance test system is shown in Figure 12. It consists of a vibration table (DataPhysics DP901, DataPhysics, San Jose, CA, USA), power supply (RIGOL DP832A, RIGOL, Suzhou, China), an FPGA generating the control clock (Xilinx XC7A35TFGG484-2, Xilinx, San Jose, CA, USA), an oscilloscope (RIGOL MSO8104, RIGOL, Suzhou, China), dynamic signal analyzer (Agilent 35670A, Agilent Technologies, Santa Clara, CA, USA) and the proposed chip. The proposed accelerometer chip is wire-bonded to a test PCB and firmly attached to the vibration table with a rigid fixture to ensure efficient transmission of vibrational excitation. A sinusoidal vibration signal with a frequency of 100 Hz and an amplitude of 0.5 g is applied via the vibration controller. The accelerometer output is sampled and recorded using an oscilloscope, followed by measurement of the dynamic signal output spectrum using a dynamic signal analyzer.

**Figure 13:**
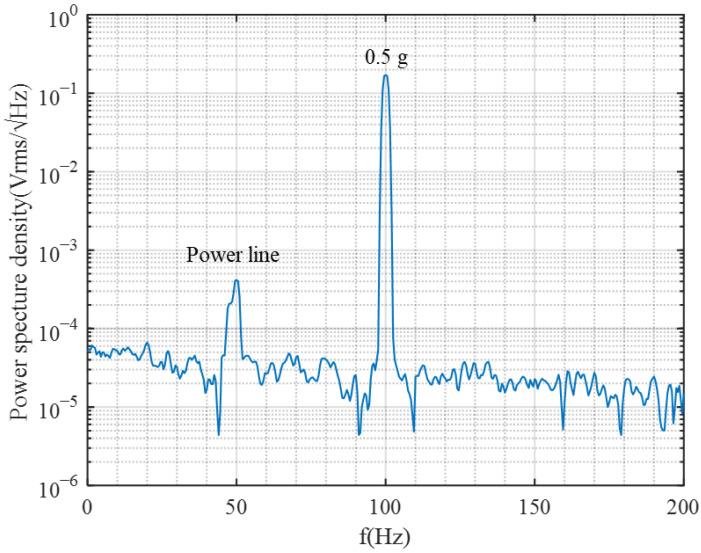
Measured spectrum of the sensing axis under a 100 Hz 0.5 g sinusoidal acceleration.

Figure 13 shows the measured spectrum of the sensing axis under a 100 Hz, 0.5 g sinusoidal acceleration. The study of the obtained spectrum reveals a peak-to-noise ratio of 88 dB between the 100 Hz acceleration signal and the surrounding system noise, indicating a dynamic range of 88 dB. The calculations determine the system’s equivalent input noise floor to be 14 $\mu g/\sqrt{Hz}$. This metric denotes the minimum acceleration input the system can discern within a unit bandwidth, serving as an essential parameter for evaluating ultimate precision and weak-signal detection capacity.

## 5. Discussion and Conclusions

Table 4 summarizes the overall actual test performance of the AFE designed in this paper. It also compares with existing designs in the literature designs. The AFE readout circuit described in this paper exhibits excellent sensitivity and linearity, low power consumption, and low noise.

An AFE readout circuit for capacitive accelerometers was designed, fabricated, and experimentally validated to meet the requirements of OIS applications. Static testing revealed a sensitivity of 342 mV/G and favorable nonlinearity characteristics. During dynamic testing, the chip demonstrated a dynamic range of 88 dB and a noise level of 14 $\mu g/\sqrt{Hz}$. The low-noise substrate enables precise detection of subtle vibrations, improving the accuracy of image stabilization corrections and enhancing overall image clarity.

In conclusion, this paper presents a low-noise AFE circuit for an MEMS capacitive accelerometer intended for OIS applications. The contribution of this work lies in the application-driven co-design of the readout architecture and circuit parameters for the target sensor and system constraints. Based on this approach, the proposed AFE achieves a measured sensitivity of 342 mV/g, nonlinearity of 1.1%, dynamic range of 88 dB, and noise floor of 14 $\mu g/\sqrt{Hz}$. The experimental results support the effectiveness of the proposed readout strategy for compact OIS applications.

An important direction for future development is the monolithic integration of the MEMS accelerometer and its interface electronics on a single chip. The current work employs a hybrid approach, with the SOG MEMS sensor and CMOS AFE fabricated separately and then co-packaged. While this approach has enabled rapid prototyping and independent optimization of the mechanical and electrical components, monolithic integration offers several potential advantages. First, monolithic integration can eliminate bond wires and interconnects between the sensor and AFE, which would minimize parasitic capacitance at the sensing nodes. Second, monolithic integration could further reduce the overall footprint, enabling even more compact camera modules for smartphones. Third, wafer-level integration and testing could reduce assembly costs for high-volume production. Nevertheless, several challenges must be addressed to realize such integration. MEMS fabrication typically requires specific structural layers and release etching steps that may not be compatible with standard CMOS processing. Solutions include pre-CMOS integration with MEMS, inter CMOS-MEMS integration and post CMOS-MEMS integration [35], each involving trade-offs in process complexity, thermal budget, contamination control, residual stress, and yield.

The presented chip already satisfies the requirements for OIS systems, with its low-noise characteristics supporting applications in handheld imaging device, drone gimbal stabilization, and automotive imaging systems. Building on this foundation, future work will explore two directions: based on the low-noise AFE design proposed in this study, the next step will be to further develop a fully integrated CMOS-MEMS accelerometer. Subsequently, we plan to enhance system-level integration and conduct closed-loop validation of the sensor with micro-controllers and OIS control modules.

## Figures and Tables

**Table 1:** Key performance requirements for acceleration sensors in OIS applications.

Performance Parameters	Design Specification
Noise level	$<32\;\mu g/\sqrt{Hz}$
Bandwidth	0–200 Hz
Acceleration range	−1 g–1 g
Die footprint	<1 ${mm}^{2}$

**Table 2:** Parameters of the accelerometer.

Parameters	Values
Technology	Silicon on glass (SOG)
Overall size	1000 μm × 950 μm
Structure thickness	45 μm
Comb fingers	120 μm × 4 μm
Resonant frequency	2050 Hz
Sensing capacitance	480 fF
Sensing range	−1 g–1 g
Mechanical sensitivity	59 nm/g
Brownian noise	$9.2\;\mu g/\sqrt{Hz}$

**Table 3:** Simulated power breakdown of the proposed AFE at 1.8 V apply.

Block	Power (mW)	Percentage (%)
First-stage amplifier	1.045	29.3
Second-stage amplifier	0.475	13.3
Chopper switches and buffer	0.32 × 4	8.9 × 4
Bias and cmfb circuits	0.136	3.8
Filters	0.636	17.8
Total	3.572	100

**Table 4:** Comparison of previously reported MEMS accelerometers.

References	Technology	Sensing Range (g)	Power Dissipation (mW)	Noise Floor $(\mu g/\sqrt{Hz}$)	Nonlinearity (%)	Chip Area
This work	SOG + CMOS	±1	3.6	14	1.1	1000 μm× 950 μm (SOG) 945 μm× 600 μm (CMOS)
Wu [10]	CMOS-MEMS	±6	30	50	N/A	600 μm× 450 μm (MEMS) 3.5 mm × 2.5 mm (total)
Tan [32]	CMOS-MEMS	±2	2.57	54	1.28	400 μm× 400 μm (MEMS) 2.38 mm × 2.38 mm (total)
Fang [33]	CMOS-MEMS	±8	1	50	0.1	N/A
Qu [11]	CMOS-MEMS	±1	1	X,Y axis:12 Z axis:110	N/A	1.23 mm × 1.23 mm (MEMS) 3 mm × 3 mm (total)
Sun [25]	CMOS-MEMS	±1	1	50	N/A	3 mm × 3 mm (total)
Baschirotto [34]	MEMS + CMOS	±1	45	13.5	N/A	2.60 mm × 2.38 mm (total)

## Data Availability

The original contributions presented in this study are included in the article. Further inquiries can be directed to the corresponding author.
